# Educators’ knowledge of and attitudes towards attention deficit hyperactivity disorder

**DOI:** 10.4102/jcmsa.v4i1.305

**Published:** 2026-03-18

**Authors:** Lesiba L.C. Mello, Dennilee Naicker, Deborah van der Westhuizen

**Affiliations:** 1 Department of Psychiatry, Faculty of Health Sciences, University of Pretoria, Pretoria, South Africa

**Keywords:** attention deficit hyperactivity disorder, educators, knowledge, attitude, child development

## Abstract

**Background:**

Attention deficit hyperactivity disorder (ADHD) is a prevalent neurodevelopmental disorder. Educators play a crucial role in identifying and referring children with ADHD symptoms and integrating them into academic settings. It is important that educators have a positive attitude and adequate knowledge of the condition. This study investigated educators’ knowledge and attitudes towards children with ADHD.

**Methods:**

This cross-sectional analytical study was conducted at mainstream private and public schools in the Tshwane district, Gauteng, South Africa. One hundred educators, comprising 63 from public and 37 from private schools, participated in the study. Each participant completed a modified demographic questionnaire and the ADHD-specific Knowledge and Attitudes of Educators scale, which included the Scale for ADHD-Specific Knowledge (SASK) and the Scale of ADHD-Specific Attitude.

**Results:**

Educators had a high level of knowledge of ADHD (mean SASK score = 70%). Private school educators demonstrated significantly higher knowledge (median SASK score = 75%) than public school educators (median SASK score = 65%). Attitudes towards ADHD-associated behaviours were unfavourable. Attitude did not correlate with knowledge (*p* = 0.57) or teaching experience (*p* = 0.21), nor did it differ between public and private school educators (*p* = 0.62).

**Conclusion:**

In this study, educators had adequate knowledge of ADHD and acknowledged it as a valid diagnosis. Despite this, they reported negative personal experiences and challenges in managing ADHD-type behaviours in the classroom. Educators remained committed to supporting learners and highlighted a critical need for effective classroom management strategies and training.

**Contribution:**

This study offers actionable insights for improving support for learners with ADHD and their educators.

## Introduction

Attention deficit hyperactivity disorder (ADHD) is one of the most common psychiatric disorders in childhood, with a global prevalence estimated at 7.2%,^1^ consistent with findings in sub-Saharan Africa.^2^ Attention deficit hyperactivity disorder is a neurodevelopmental disorder characterised by developmental deficits and changes in brain function beginning in early childhood. Recent dimensional approaches to measuring ADHD symptoms encompass a broad range of severity but lack clear boundaries distinguishing the condition from typical development.^1,3^

A diagnosis of ADHD requires a persistent pattern of inattention, hyperactivity or impulsivity severe enough to impair academic functioning in school-aged children in structured environments.^1^ Symptoms often become more apparent at school, where demands on self-regulation are high.^4^ In addition to academic difficulties, children with ADHD frequently experience strained relationships with peers and family, higher rates of suspension or expulsion and comorbid psychiatric conditions,^5^ all of which can contribute to long-term challenges if unaddressed.^6^

Educators play a critical role in identifying, assessing and referring children who exhibit ADHD-type behaviour.^7^ Educators’ perceptions of ADHD are shaped by their knowledge of ADHD,^7^ which correlates with their attitudes and classroom practices.^8,9^ Therefore, educators must possess accurate knowledge of ADHD and positive attitudes to support and refer students who need assistance.^10^

A study conducted in the Caribbean found that educators in Trinidad and Tobago had below-average knowledge of ADHD, with those holding master’s degrees or prior training scoring highest.^11^ This finding aligns with a cross-national study conducted across nine countries (the Czech Republic, Germany, Greece, Iraq, Vietnam, Saudi Arabia, the United States, South Africa and the Republic of Korea), which showed that while knowledge levels varied greatly, exposure to ADHD and training consistently improved understanding.^12^

In South Africa, studies on educators’ knowledge of ADHD have produced mixed results. Some studies reported average to above-average knowledge, especially regarding symptoms,^13,14^ while others highlighted significant gaps.^7,15,16^ Studies showing limited knowledge typically had larger samples and included public sector schools.^14^ Educators in private schools were generally better informed,^17^ likely because of more in-service training.^14^ A study in Soweto found that educators’ knowledge of ADHD did not vary by years of experience,^16^ contrasting with findings by Sciutto et al.,^12^ who reported that prior experience teaching students with ADHD was associated with higher knowledge levels.

Research indicates that educators often hold negative attitudes towards children exhibiting ADHD-type behaviours.^10,18^ Prior exposure to such children has been linked to less tolerance, suggesting that such attitudes may become more negative over time.^10^ This aligns with the findings that younger educators have more positive attitudes towards children with ADHD in their classrooms.^19^ Yarde-Leavett^18^ reported that experienced and knowledgeable educators generally have more positive attitudes and a willingness to learn about ADHD. Conversely, limited knowledge can lead to stereotyping children with ADHD as simply needing stricter discipline.^16^

The recognition of ADHD is growing, and educators are increasingly serving as key figures in early identification and support. In South Africa, few studies have examined educators’ knowledge and attitudes towards ADHD. This study addresses this gap using the ADHD-specific Knowledge and Attitudes of Educators (ASKAT) scale to assess educators in the Tshwane district, Gauteng, South Africa, an under-researched area. Secondary objectives included comparing private schools with public schools, secondary schools with primary schools and exploring how teaching experience influences attitudes.

## Research methods and design

### Study design

This study was a quantitative cross-sectional analytical study. Data were collected at a single point in time using a self-administered online survey to investigate and compare levels of knowledge and attitudes towards ADHD among educators within the Tshwane district, Gauteng, South Africa.

### Setting

This study was conducted in public and private schools across three Tshwane districts (North, West and South) in Gauteng province, South Africa. These districts and school types were selected to capture diverse socio-economic and educational backgrounds.

### Study population and sampling strategy

The study aimed to include 60 private schools and 120 public schools. A minimum sample of 90 participants was calculated to provide 86 degrees of freedom for residual mean squares, exceeding the convention of at least 30 degrees of freedom. Convenience sampling was employed. One hundred educators participated in the study, including 63 from public schools and 37 from private schools. All questionnaires were completed in full and included in the analysis. Eligible participants were educators aged 18 years or older, able to provide informed consent, proficient in English and currently employed at a primary or secondary school in the Tshwane district, Gauteng, South Africa. Individuals not meeting these criteria were excluded. Participation was voluntary.

### Data collection

Data were collected over a six-month period, from February to August 2024. The study used the ASKAT questionnaire, comprising four subsections and two quantitative subscales: the Scale for ADHD-Specific Knowledge (SASK) and the Scale for ADHD-Specific Attitudes (SASA).^20^ The quantitative scales were combined with a modified demographic questionnaire that collected information such as participants’ age, gender, educational attainment, years of teaching experience, school type (public or private) and whether they had taught learners exhibiting ADHD-type behaviours. The SASK consists of 20 items measuring educators’ ADHD-specific knowledge, with ‘true’, ‘false’ and ‘I don’t know’ response options. Scale for ADHD-Specific Attitudes includes 30 Likert-scale items assessing cognitive beliefs, affective states and perceived control, with responses ranging from one to six. The ASKAT scales are derived primarily from diagnostic and statistical manual of mental disorder-5 (DSM), international classification of disease (ICD)-10 and seminal sources, making them most applicable in English-speaking settings that utilise the DSM-5 framework.^20^ However, their validity for use in the South African context has not been established. We obtained permission to use the scale. An online survey containing the ASKAT and modified demographic questionnaire was distributed via school administrators for voluntary completion. Educators completed the 15 min – 20-min survey using Microsoft Forms.

### Data analysis

Correct response and frequency analyses were used to evaluate educators’ knowledge about ADHD. An exploratory factor analysis (EFA) was conducted on the attitude data to examine the underlying structure of the items. Means, standard deviations, medians and interquartile ranges were described for continuous variables. The Shapiro–Wilk test was used to assess whether continuous data, such as age, followed a normal distribution. Categorical variables were described using proportions and frequencies.

The Kruskal–Wallis test, Wilcoxon two-sample test and Spearman correlation coefficients were used to compare groups and assess associations for non-parametric variables. Findings were deemed significant if *p* < 0.05.

### Ethical considerations

This study was approved by the Faculty of Health Sciences Research Ethics Committee at the University of Pretoria (Reference: 457/2023). The Gauteng Department of Education granted additional authorisation (Reference: 2024/515), as well as the relevant school districts and principals. Prospective participants received an information leaflet outlining the study. Completion and submission of the survey served as confirmation of consent. Confidentiality was protected throughout the study.

## Results

Participant characteristics are presented in Table 1. One hundred educators participated in the study, with 76% identifying as female and 24% as male. According to the Shapiro–Wilk test for normality, participant age did not follow a normal distribution (*W* = 0.800, *p* < 0.0001). Therefore, the median age was 39.5 years (interquartile range [IQR]: 31–54.5).

**TABLE 1:** Demographic characteristics of educators (*N* = 100) who completed the attention deficit hyperactivity disorder-specific knowledge and attitudes of educators scale and demographic survey.

Variable	Categories	*n*	%	Median	Interquartile range	
					Q1	Q3
Age (years)						
	All	100	100	39.5	31.0	54.5
	20–29	19	19	-	-	-
	30–39	31	31	-	-	-
	40–49	19	19	-	-	-
	50–59	25	25	-	-	-
	60–69	6	6	-	-	-
Gender						
	Female	76	76	-	-	-
	Male	24	24	-	-	-
Educational level						
	Degree	51	51	-	-	-
	Diploma	10	10	-	-	-
	Postgraduate	39	39	-	-	-
School type						
	Private	37	37	-	-	-
	Public	63	63	-	-	-
Teaching experience						
	All	100	100	11.0	6.0	22.5
	0–5 years	24	24	66.4	63.6	68.9
	6–10 year	24	24	63.6	62.5	66.1
	11–20 years	26	26	65.0	59.4	67.8
	21–30 years	14	14	65.8	63.3	71.1
	31–40 years	12	12	61.4	58.1	66.7
Teaching child exhibiting ADHD-type behaviour						
	Yes	92	92	-	-	-
	No	8	8	-	-	-
No: of students with ADHD						
	ADHD	53	57.1	-	-	-
	No ADHD	39	42.39	-	-	-
ADHD a legitimate educational problem						
	Yes	89	89	-	-	-
	No	5	5	-	-	-
	Unsure	4	4	-	-	-
	Sometimes	1	1	-	-	-
	Yes & No	1	1	-	-	-
In-service training						
	Yes	21	21	-	-	-
	No	79	79	-	-	-

ADHD, attention deficit hyperactivity disorder.

Participants varied in educational attainment: 51% held undergraduate degrees, while 31% had completed postgraduate qualifications. The median teaching experience was 11 years (IQR: 6–22.5). Of the participants, 37% taught in private schools and 63% in public schools.

Ninety per cent of educators reported teaching children exhibiting ADHD-type behaviour, and 57.6% of these educators verified that some learners had received a formal diagnosis. Common challenges included a lack of discipline, disruptive classroom behaviour, poor participation and difficulty maintaining concentration. Overcrowded classrooms and limited training further impeded educators’ ability to effectively support and accommodate these learners.

### Knowledge

The SASK results are summarised in Table 2. On average, educators answered 70% (IQR: 57.5–80) of the questions correctly, demonstrating adequate knowledge of ADHD. The SASK tested four domains. Educators were most knowledgeable about symptom-related questions, with an average accuracy of 85.4%, followed by questions on prevalence and assessment (80.5%). Knowledge of aetiology was lower, with an average score of 59.2%. Participants performed particularly poorly on treatment-related items: 94% incorrectly believed that special diets are an effective treatment for ADHD, resulting in an average score of 50.5% in this category.

**TABLE 2:** Educators’ knowledge of attention deficit hyperactivity disorder.

SASK item	Correct		Incorrect	
	*n*	%	*n*	%
Q1: ADHD is a neurobiological, developmental disorder.	74	74	26	26
Q2: Special diets (e.g. reduced sugar, wheat free, lactose free, additive free) are an effective treatment for ADHD.	6	6	94	94
Q3: Children with ADHD tend to have poor concentration.	87	87	13	13
Q4: A combination of stimulant medication and behaviour management is an effective treatment for ADHD.	78	78	22	22
Q5: There are different subtypes of ADHD which can present with different behaviours	84	84	16	16
Q6: ADHD can be inherited	62	62	38	38
Q7: Children with ADHD can present with hyperactive behaviours, inattentive behaviours, or a combination of both.	91	91	9	9
Q8: Children with ADHD are easily distracted.	97	97	3	3
Q9: Children with ADHD benefit from stricter parenting and schooling.	34	34	66	66
Q10: ADHD is caused by too much sugar in the diet.	47	47	53	53
Q11: Children who have the hyperactive type of ADHD often talk excessively and have dificulty staying in their seat.	91	91	9	9
Q12: ADHD is caused by poor parenting.	75	75	25	25
Q13: Children with ADHD can choose to be better behaved	63	63	37	37
Q14: Some children can present with inattentive or hyperactive behaviours yet not meet the criteria for an ADHD diagnosis.	75	75	25	25
Q15: There is approximately one child in every class that exhibits ADHD type behaviours.	85	85	16	16
Q16: Children with ADHD often have problem concentrating on table-top work.	100	100	0	0
Q17: Children with ADHD often fail to give close attention to their work and make careless mistakes	88	88	12	12
Q18: Teachers are often the first to recognise ADHD type behaviours and refer children for assessment.	77	77	23	23
Q19: The cause of ADHD is unknown.	38	38	62	62
Q20: Children who present with ADHD behaviours, regardless of ADHD diagnosis, can benefit from individualised behaviour management strategies.	84	84	16	16
**Total**	**70**	**70**	**30**	**30**

Private school (*n* = 37) and public school (*n* = 63).

ADHD, attention deficit hyperactivity disorder; SASK, Scale for ADHD-Specific Knowledge.

**FIGURE 1:**
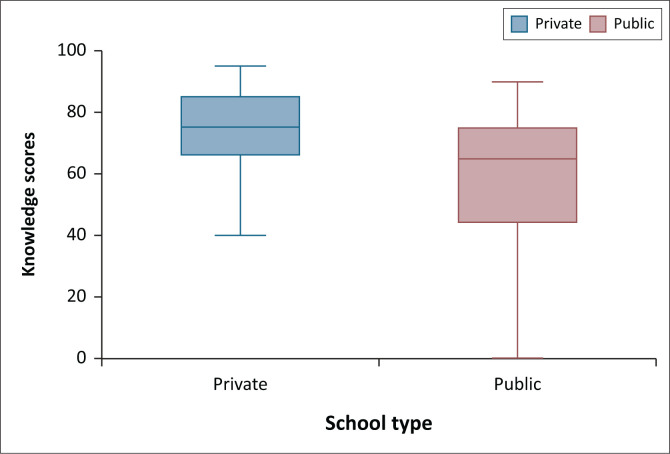
Boxplot (median ± min, max) of total knowledge scores of all private school educators (*n* = 37) and public school educators (*n* = 63) who completed the scale for attention deficit hyperactivity disorder-specific knowledge.

Educators from private schools scored a median of 75% (IQR: 70.0–85.0) on the SASK, compared to 65% (IQR: 50–75) for public school educators, indicating significantly higher knowledge among private school educators (*p* < 0.0001; Figure 1). Among all educators, 33% reported receiving training on ADHD, including 48% of private school educators and 29% of public school educators.

### Attitudes

#### Frequency analysis

The SASA results are presented in Table 3. Most educators rejected negative stereotypes: 93% affirmed that ADHD is a valid diagnosis, and fewer than half believed it is over-diagnosed. Notably, 78% did not believe that children with ADHD-associated behaviours are deliberately misbehaving.

**TABLE 3:** Attitudes of educators towards attention deficit hyperactivity disorder (*N* = 100).

Scale of ADHD-Specific altitude questions	Percentage					
	Strongly disagree	Disagree	Somewhat disagree	Somewhat agree	Agree	Strongly agree
**Q1: Students who exhibit behaviors associated with ADHD**						
Q1.1 Are rewarding to work with	3	4	9	11	32	41
Q1.2 Interfere with my ability to teach my class	5	8	1	10	32	44
Q1.3 Perform well in some subjects and not others	5	1	2	6	42	44
Q1.4 Have no excuse for their behaviour if they do not have a formal diagnosis	24	50	15	7	4	0
Q1.5 Misbehave because they don’t want to follow a set of rules	25	41	27	1	5	1
Q1.6 Need more structure and discipline, not assistance with their acadernic work	20	37	24	11	6	2
Q1.7 Bring new perspectives to the topic I am teaching	0	4	2	27	49	18
Q1.8 Need to try harder to focus on their schoolwork	13	40	18	8	14	7
**Q2: I believe**						
Q2.1 ADHD is over diagnosed	7	21	16	29	20	7
Q2.2 ADHD is a valid diagnosis	1	3	3	21	49	23
Q2.3 ADHD is an excuse for poor parenting	51	24	12	6	5	2
Q2.4 Children who exhibit ADHD type behaviour are deliberately misbehaving.	35	30	13	11	8	3
**Q3: I would**						
Q3.1 Refer a student who exhibited ADHD-type behaviours in my classroom to the school counsellor for possible assessment	5	2	1	15	36	41
Q3.2 Like to know more about ADHD and associated behaviours	4	2	1	9	40	44
Q3.3 Like to have more information about classroom interventions to assist me with educating students who display ADHD-type behaviours	3	1	2	6	41	47
**Q4: I find**						
Q4.1 Behaviours associated with ADHD irritating in the classroom	7	10	12	30	27	14
Q4.2 Students who exhibit ADHD-type behaviours rude	7	25	15	27	19	7
Q4.3 It challenging to teach students who exhibit behaviours associated with ADHD	2	4	10	28	36	20
Q4.4 It rewarding to see accomplishments of students who display ADHD-type behaviours	3	4	7	8	46	32
Q4.5 Students who display ADHD-type behaviours cause me to experience stress	4	11	12	29	30	14
**Q5: When it comes to differentiation, I feel**						
Q5.1 ADHD is a benefit to the growth of my teaching skills	6	5	8	19	44	18
Q5.2 I don’t have time	13	25	15	25	13	9
Q5.3 I already change my lessons and teaching styles	2	7	10	39	32	10
Q5.4 Educational accommodations for students with ADHD-type behaviours are easy to implement in a general education classroom	6	24	25	19	13	13
**Q6: I feel am knowledgeable about**						
Q6.1 ADHD-type behaviour	8	24	13	29	19	7
Q6.2 Classroom interventions to manage misbehaviour	7	11	8	29	34	11
**Q7: I feel**						
Q7.1 I have received adequate professional development about managing ADHD-type behaviour	17	37	13	18	11	4
Q7.2 I can effectively teach students who exhibit behaviours associated with ADHD	6	22	11	34	23	4
Q7.3 I want to be more effective teaching students who display ADHD-type behaviours	2	4	3	11	46	34
Q7.3 I dislike teaching classes that contain students who display ADHD type behaviours	20	30	16	22	8	4

ADHD, attention deficit hyperactivity disorder.

Educators generally agreed with positive statements about working with students who exhibit ADHD-type behaviour. For example, 84% of participants agreed that ‘students who exhibit behaviours associated with ADHD are rewarding to work with’, and 81% believed that ‘students who exhibit behaviours associated with ADHD bring new perspectives to the topics’.

Despite positive views about the children, most educators reported negative attitudes towards ADHD-type behaviours. Seventy-three per cent agreed that ‘students who display ADHD-type behaviours cause them to experience stress’, and 71% found ‘behaviours associated with ADHD irritating in the classroom’. Although many educators felt competent teaching students with ADHD, 67% reported that they had not received adequate professional training. Additionally, 55% stated that ‘accommodations for students with ADHD-type behaviour are not easy to implement in a general education classroom’. There was no significant difference in median SASA scores between public and private schools. However, some item-level differences emerged. For example, regarding the statement that learners who exhibited certain behaviours have no excuse for their poor behaviour if they did not have a formal diagnosis, 100% of private school educators disagreed, while 89% of public school educators disagreed with the statement.

A weak negative correlation was observed between SASK and SASA scores (*r* = −0.06), and no significant association was found between knowledge and attitudes (*p* = 0.5706). Attitudes towards ADHD, as measured by SASA percentage scores, were compared across five groups of teaching experience using the Kruskal–Wallis test. No statistically significant difference in median attitude scores was found between the groups (*p* = 0.217).

Because of the imbalance in the data, primary and secondary schools were not compared to avoid biased or invalid conclusions.

#### Exploratory factor analysis

**FIGURE 2:**
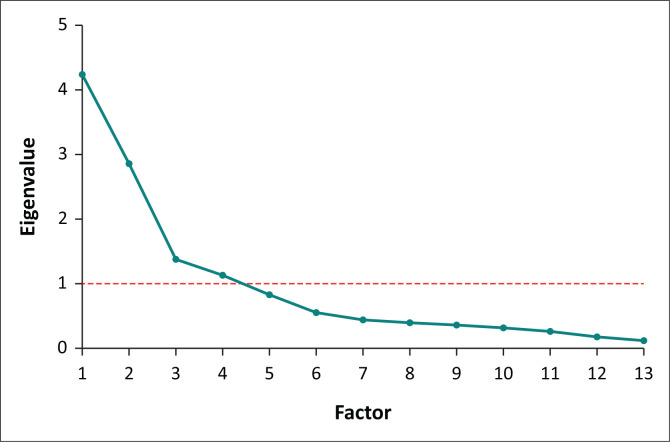
Scree plot of factor extraction.

An EFA was performed on the 30-item SASA to examine the underlying constructs shaping educators’ attitudes. Prior to extraction, the data were confirmed to be suitable for factor analysis. A four-factor structure was extracted. The scree plot and factor eigenvalues were used to evaluate whether the structure was adequate. The four-factor structure was corroborated by a distinct break in the scree plot, as shown in Figure 2, and all four factors had eigenvalues greater than one. Collectively, these factors accounted for 73.52% of the total variance, exceeding the recommended 60% threshold for a satisfactory solution.^21^

The factors were labelled as follows: (1) desire for knowledge and skills, (2) educator self-efficacy and training, (3) perceptions of student impact and support and (4) negative affective responses to ADHD behaviours. Standardised factor loadings were interpreted as meaningful if they exceeded 0.30.^21^ Nearly all loadings were above the conventional 0.40 threshold, with only one loading slightly below at 0.355. The items, their factor loadings, eigenvalues, percentage of variance explained and Cronbach’s alpha coefficients for the four-factor solution are detailed in Table 4.

**TABLE 4:** Factor loadings for exploratory factor analysis of the scale of attention deficit hyperactivity disorder-specific attitude (*N* = 100).

Items	Factor 1	Factor 2	Factor 3	Factor 4
**Factor 1: Desire for knowledge and skills**				
I would like to have more information about classroom interventions	0.94	-	-	-
I would like to know more about ADHD and its associated behaviours	0.75	-	-	-
I want to be more effective teaching students who display ADHD-type behaviours	0.67	-	-	-
**Factor 2: Educator self-efficacy and training**				
I can effectively teach students who exhibit behaviours associated with ADHD	-	0.80	-	-
I have received adequate professional development about managing ADHD-type behaviours	-	0.78	-	-
I feel I am knowledgeable about classroom interventions to manage misbehaviour	-	0.71	-	-
I feel I am knowledgeable about ADHD-type behaviours	-	0.58	-	-
**Factor 3: Perceptions of student impact and support**				
Students who exhibit behaviours associated with ADHD are rewarding to work with	-	-	0.81	-
Students who exhibit behaviours associated with ADHD interfere with my ability to teach	-	-	0.74	-
Students who exhibit behaviours associated with ADHD perform well in some subjects	-	-	0.61	-
I would refer a student for a possible ADHD assessment	-	-	0.53	-
When it comes to differentiation I think ADHD is a benefit to my teaching skill	-	-	0.33	-
**Factor 4: Negative affective responses to ADHD behaviours**				
I find students who exhibit ADHD-type behaviours rude	-	-	-	0.70
I dislike teaching classes that contain students who display ADHD-type behaviours	-	-	-	0.61
I find it challenging to teach students who exhibit behaviours associated with ADHD	-	-	-	0.59
I find behaviours associated with ADHD irritating in the classroom	-	-	-	0.58
Students who display ADHD-type behaviours cause me to experience stress	-	-	-	0.49
I believe children who exhibit ADHD-type behaviours are deliberately misbehaving	-	-	-	0.44
Eigenvalues	4.22	2.85	1.37	1.13
% of variance	32.43	21.89	10.53	8.67
Cronbach’s α	0.85	0.81	0.77	0.74

ADHD, attention deficit hyperactivity disorder.

## Discussion

In this study, educators demonstrated adequate knowledge of ADHD, achieving an average score of 70% on the ASKAT. This performance was notably higher than that reported in several South African studies, where participants averaged below 60%, as well as in some international studies with similarly low scores.^7,11,12,15,17,22^ Participants performed best on questions related to symptomatology and least well on treatment-related items, consistent with Mulholland et al.’s findings that educators are generally more knowledgeable about symptoms.^10^ Additionally, most educators (94%) believed that diet is an effective treatment for ADHD, reflecting a widespread misconception frequently documented in the literature.^7^

Similar to the current study, research conducted in Johannesburg, South Africa, found that educators in private schools were more knowledgeable about ADHD than their counterparts in public schools, attributing this to greater access to in-service training.^17^ This pattern was also evident here, as 48% of private school educators reported receiving ADHD training compared to 29% of public school educators.^17,23^ To emphasise this point, a study in Tshwane assessing pre- and post-training knowledge among educators indicated that semi-rural schools had less knowledge than urban schools before training, but these differences disappeared after training.^24^

Educators in this study generally expressed negative perceptions of ADHD-type behaviours, with most describing them as irritating and stressful in the classroom. Furthermore, 84% of educators reported that teaching children who display such behaviour is challenging, a finding consistent with previous studies.^18,20^ Educators also identified overcrowded classrooms, limited resources and a lack of training as factors that restricted their ability to meet learners’ needs, contributing to frustration. These concerns were echoed by educators in a 2021 study conducted in Soweto.^16^

Encouragingly, most of the educators in this study believed that ADHD was a valid diagnosis and a legitimate educational concern. Although knowledge levels varied between private and public schools, these differences did not affect educators’ beliefs in the validity of the diagnosis. This finding aligns with research conducted in Trinidad and Tobago.^11^ Despite these challenges, most educators expressed positive sentiments about working with children with ADHD, noting that such experiences are rewarding and contribute to their professional development, consistent with other studies.^10,23^

Although many participants had extensive exposure to learners with ADHD and varying teaching experience, we found no significant association between experience and attitudes. This contrasts with studies in Australia, which reported that educators with more experience and knowledge tended to have more positive attitudes.^25,26^

Educators also expressed a willingness to refer students displaying ADHD-type behaviours for further assessment and endorsed combining stimulant medication with behavioural interventions as an effective management approach, similar to findings in Yarde-Leavett’s^18^ study.

No significant correlation emerged between knowledge and attitudes in this study. However, gaps in understanding remain, particularly concerning classroom management and persistent misconceptions about diet and parenting. Most educators have a strong desire for better ADHD training and classroom interventions to support learners with ADHD. This finding aligns with previous studies,^18,19^ underscoring the importance of targeted professional development.

This study had several limitations. The results cannot be generalised beyond Tshwane, Gauteng, South Africa, as the unique socio-economic and educational environment in Tshwane may differ from conditions elsewhere. The exclusive use of quantitative data limited deeper exploration of educators’ experiences and perceptions. Additionally, the imbalance in sample sizes between public and private schools prevented meaningful comparisons. Finally, some schools may not have responded because of the use of multiple contact details during recruitment.

## Conclusion

This study supports existing evidence that educators’ knowledge of ADHD is generally adequate, with private school educators demonstrating higher levels of knowledge than those in public schools. Unlike many other studies, we did not find a correlation between knowledge and attitudes. While educators held generally favourable attitudes towards the diagnosis of ADHD and children with the condition, their personal attitudes towards managing ADHD-type behaviours in the classroom were unfavourable. Educators also reported that working with these students was rewarding and expressed a desire to develop more effective classroom management strategies. Practical actions are required to convert knowledge into efficient practice. We suggest a dual approach focusing on the provision of easily accessible classroom management resources and required practical in-service training. Such investments are critical to reduce educator frustration, improve classroom environments and empower learners with ADHD to thrive both academically and socially.

## References

[1] American Psychiatric Association, First MB. Diagnostic and statistical manual of mental disorders: DSM-5-TR. 5th ed. Text revision ed. Washington, DC: American Psychiatric Association Publishing; 2022.

[2] Bakare MO. Attention deficit hyperactivity symptoms and disorder (ADHD) among African children: A review of epidemiology and co-morbidities. Afr J Psychiatry. 2012;15(5):358–361. 10.4314/sajpsy.v15i5.45 23044891

[3] Sonuga-Barke E, Thapar A. The neurodiversity concept: Is it helpful for clinicians and scientists? Lancet Psychiatry. 2021;8(7):559–561. 10.1016/S2215-0366(21)00167-X 33984295

[4] Schwean VL, Parkinson M, Francis G, Lee F. Educating the ADHD child: Debunking the myths. Can J Sch Psychol. 1993;9(1):37–52. 10.1177/082957358500900105

[5] Barkley RA. Behavioral inhibition, sustained attention, and executive functions: Constructing a unifying theory of ADHD. Psychol Bull. 1997;121(1):65–94. 10.1037/0033-2909.121.1.65 9000892

[6] Mash EJ, Wolfe DA. Abnormal child psychology. 7th edn. Belmont, CA: Cengage Learning; 2019.

[7] Topkin B, Roman NV, Mwaba K. Attention deficit disorder (ADHD): Primary school teachers’ knowledge of symptoms, treatment and managing classroom behaviour. S Afr J Educ. 2015;35(2):1–8. 10.15700/saje.v35n2a988

[8] Kos JM, Richdale AL, Hay DA. Children with attention deficit hyperactivity disorder and their teachers: A review of the literature. Int J Disabil Dev Educ. 2006;53(2):147–160. 10.1080/10349120600716125

[9] Glass CS, Wegar K. Teacher perceptions of the incidence and management of attention deficit hyperactivity disorder. Education. 2000;121(2):412.

[10] Mulholland SM, Cumming TM, Jung JY. Teacher attitudes towards students who exhibit ADHD-type behaviours. Aust J Spec Educ. 2015;39(1):15–36. 10.1017/jse.2014.18

[11] Youssef MK, Hutchinson G, Youssef FF. Knowledge of and attitudes toward ADHD among teachers: Insights from a Caribbean nation. Sage Open. 2015;5(1):21582440145. 10.1177/2158244014566761

[12] Sciutto MJ, Terjesen MD, Kučerová A, et al. Cross-national comparisons of teachers’ knowledge and misconceptions of ADHD. Int Perspect Psychol. 2016;5(1):34–50. 10.1037/ipp0000045

[13] Jaye TH, Levy C, Majakwara J, Hanson S. Foundation Phase teachers’ understanding of attention deficit hyperactivity disorder at Johannesburg independent schools. S Afr J Child Educ. 2020;10(1):1–11. 10.4102/sajce.v10i1.825

[14] Sim G. Assessing attention deficit hyperactivity disorder (ADHD) specific knowledge in educators and identifying demographic predictors pertaining to educators’ knowledge of ADHD within the South African context [thesis]. Durban: University of KwaZulu-Natal, Howard College Campus; 2021 [cited 2023 May 15]. Available from: https://researchspace.ukzn.ac.za/handle/10413/20735

[15] Perold M, Louw C, Kleynhans S. Primary school teachers’ knowledge and misperceptions of attention deficit hyperactivity disorder (ADHD). S Afr J Educ. 2010;30(3):457–473. 10.15700/saje.v30n3a364

[16] Maema EK. Grade three teachers’ experiences of learners perceived to have ADHD in Soweto mainstream primary schools [dissertation]. Pretoria: University of South Africa; 2021 [cited 2023 Mar 31]. Available from: https://hdl.handle.net/10500/27265

[17] Kern A, Amod Z, Seabi J, Vorster A. South African foundation phase teachers’ perceptions of ADHD at private and public schools. Int J Environ Res Public Health. 2015;12(3):3042–3059. 10.3390/ijerph120303042 25768242 PMC4377951

[18] Yarde-Leavett C. Teachers’ knowledge of and attitudes towards ADHD in the Western Cape [master’s thesis]. Cape Town: University of Cape Town; 2018 [cited 2023 Mar 31]. Available from: https://open.uct.ac.za/handle/11427/29385

[19] Bornman J, Donohue DK. South African teachers’ attitudes toward learners with barriers to learning attention-deficit and hyperactivity disorder and little or no functional speech. Int J Disabil Dev Educ. 2013;60(2):85–104. 10.1080/1034912X.2013.786554

[20] Mulholland S. ADHD-specific knowledge and attitudes of teachers (ASKAT): Development and validation of a new research instrument. Int J Educ Res. 2016;77:109–116. 10.1016/j.ijer.2016.03.010

[21] Hair JF, Black WC, Babin BJ, Anderson RE. Multivariate data analysis: A global perspective. 7th ed. Upper Saddle River, NJ: Pearson; 2010.

[22] Etchells W. Foundation and Intermediate Phase Educators’ Knowledge and Beliefs about the Features, Symptoms and Diagnosis of Attention Deficit Hyperactivity Disorder (ADHD) [thesis]. Pietermaritzburg: University of KwaZulu-Natal; 2015 [cited 2023 May 15]. Available from: http://hdl.handle.net/10413/13002.

[23] Greenway CW, Rees Edwards A. Knowledge and attitudes towards attention-deficit hyperactivity disorder (ADHD): A comparison of teachers and teaching assistants. Aust J Learn Difficult. 2020;25(1):31–49. 10.1080/19404158.2019.1709875

[24] De Jongh M, Wium A-M. Attention deficit hyperactivity disorder: Training outcomes for Grade R teachers in an urban and semi-rural context. S Afr J Child Educ. 2021;11(1):a894. 10.4102/sajce.v11i1.894

[25] Sigal E, Sukenik N. What do you know about ADHD? A comparison between mainstream and special education teachers. Aust J Teach Educ. 2024;49(12):19–41. 10.14221/1835-517X.6217

[26] Ghanizadeh A, Bahredar MJ, Moeini SR. Knowledge and attitudes towards attention deficit hyperactivity disorder among elementary school teachers. Patient Educ Couns. 2006;63(1–2):84–88. 10.1016/j.pec.2005.09.002 16504452

